# Successful microvascular surgery in patients with thrombophilia in head and neck surgery: a case series

**DOI:** 10.1186/s13256-024-04403-8

**Published:** 2024-02-28

**Authors:** Julian Faber, Frank Schuster, Stefan Hartmann, Roman C. Brands, Andreas Fuchs, Anton Straub, Markus Fischer, Urs Müller-Richter, Christian Linz

**Affiliations:** 1 Department of Oral and Maxillofacial Plastic Surgery, University Hospital Cologne, 50937 Cologne, Germany; 2 Department of Anaesthesia and Critical Care, Donau-Isar-Klinikum, 94469 Deggendorf, Germany; 3 Department of Oral and Maxillofacial Plastic Surgery, University Hospital Würzburg, 97070 Würzburg, Germany

**Keywords:** Thrombophilia, Microvascular surgery, Free flap, Anticoagulation, Factor V Leiden mutation, Head and neck, Case report

## Abstract

**Background:**

In this case series, a perioperative anticoagulation protocol for microvascular head and neck surgery in patients with thrombophilia is presented. Microvascular free-flap surgery is a standard procedure in head and neck surgery with high success rates. Nevertheless, flap loss—which is most often caused by thrombosis—can occur and has far-reaching consequences, such as functional impairment, prolonged hospitalization, and increased costs. The risk of flap loss owing to thrombosis is significantly increased in patients with thrombophilia. Therefore, perioperative anticoagulation is mandatory. To date, no perioperative anticoagulation protocol exists for these high-risk patients.

**Case presentation:**

We present three exemplary male Caucasian patients aged 53–57 years with free flap loss owing to an underlying, hidden thrombophilia.

**Conclusion:**

We present a modified anticoagulation protocol for microvascular surgery in these high-risk patients, enabling successful microsurgical reconstruction.

## Introduction

Microvascular free-flap surgery has become a standard procedure in head and neck surgery with a very high success rate of approximately 94–96% [1]. However, each complication of the free flap procedure may lead to its failure. Consequences include permanent functional impairment, prolonged hospitalization, increased costs, and delays in adjuvant therapy—a frustrating situation for the patient as well as the surgeon [2]. The most important complication that causes flap loss is thrombosis in the pedicle—in most cases, venous and, less often, arterial [1, 3].

Despite meticulous microvascular surgery by experienced surgeons and perioperative thrombosis prophylaxis, unsalvageable pedicle thrombosis that leads to permanent flap loss can occur. One reason for such cases can be asymptomatic, and therefore unknown, thrombophilia.

The most common form of thrombophilia in Caucasians is activated protein C (APC) resistance, which occurs in 95% of cases owing to a factor V Leiden (FVL) mutation and has a relatively high prevalence of up to 10–15% [4–6]. APC usually leads to inactivation of activated coagulation factor V. Owing to a mutation in the factor V gene, the mutated factor V is not affected by APC, and the antithrombotic effect of APC cannot take place. This leads to a fivefold and a 50–100-fold relative risk of thrombosis for heterozygous and homozygous forms, respectively [4, 6].

Regarding statistic data, in our department, about ten patients per year who need microsurgery suffer from thrombophilia. However, many surgeons are unaware of the problem of thrombophilia and its resulting complications in microsurgery. Moreover, no current recommendation for management in head and neck surgery exists that addresses this clinically relevant issue.

Within a short period, three patients in our department suffered free flap loss owing to unusual, massive thromboses in the pedicle. Flap salvage was not possible. Therefore, prior to a second microvascular reconstruction, a thrombophilia diagnosis was run and a heterogeneous factor V Leiden mutation was found in all three patients.

The aim of this case series was to develop an easily applicable anticoagulation protocol to ensure successful microvascular surgery in the head and neck in these high-risk cases.

## Cases

The three presented Caucasian patients suffered from oral squamous cell carcinoma (OSCC). In all three patients, the underlying FVL mutation was unknown at the time of surgery. None of the patients suffered from a relevant general illness, such as underlying cardiac disease or diabetes, and none took medication regularly at the time of diagnosis. Patients 1 and 2 had a history of nicotine abuse; patient 1 also had a history of alcohol abuse. A relevant compromise of the vascular status was not recognizable in the preoperative imaging or intraoperatively in any case. After guideline-based staging, tumor resection, and neck dissection, an experienced surgeon performed microsurgical reconstruction using our standard perioperative anticoagulation protocol for microvascular surgery (Tables 1 and 2).

**Table 1:** Patients’ data and information

	Patient 1	Patient 2	Patient 3
Tumor stadium	pT2 pN0 L0 V0 Pn0 G2 R0	pT4 pN1 L0 V0 Pn1 G2 R0	pT4a pN0 L0 V0 Pn0 G3 R0
Tumor localization	Anterior floor of the mouth	Right mandibular region 45–48	Right alveolar ridge region 32–45
Microvascular flap	Radial forearm flap	Fibula flap	Fibula flap, radial forearm flap, another fibula flap, and scapula flap
Failed flap	Radial forearm flap	Fibula flap	Scapula flap
Postoperative day of flap failure	1	0 (intraoperative)	0 (intraoperative)
Cause of failure	Extensive thrombosis in both arterial and venous pedicle	Extensive and multiple thromboses during microsurgery	Extensive and multiple thromboses during microsurgery
Number of salvage attempts	1	3	2
Therapy	Adjuvant radiotherapy	Adjuvant combined radiochemotherapy	Adjuvant combined radiochemotherapy
Successful flap	Fibula flap	Fibula flap	Scapula flap

**Table 2:** Standard anticoagulation protocol

Timing		Drug	Dose
Preoperative	Inpatient stay	Enoxaparin subcutaneous	0.5 mg/kg bw 0–0–1
Intraoperative	Just before flap dissection	Heparin intravenous	Bolus of 1500 U
Postoperative	Intensive ward	Heparin intravenous	Goal PTT of 45 seconds
	Inpatient stay normal ward	Enoxaparin subcutaneous	0.5 mg/kg bw 1–0–1 for 7 days

*bw* bodyweight, *PTT* partial thromboplastin time

### Case report 1

The first patient was a 53-year-old Caucasian male diagnosed with a pT2 pN0 cM0 L0 V0 Pn0 G2 R0 OSCC in the anterior floor of the mouth. A radial forearm flap was used for microsurgical reconstruction. After one day, flap perfusion was compromised. Despite several salvage attempts, we had to explant the flap owing to necrosis caused by a long-segment thrombosis in both the venous and arterial pedicle. Defect closure was performed via local closure and granulation. Adjuvant radiotherapy was then conducted.

In the further course of treatment, clinical and radiologic examination unfortunately revealed signs of osteonecrosis of the mandible with a cutaneous fistula. Since an extended resection of the entire continuity of the jaw was indicated, we planned a microsurgical fibula flap transplantation.

Before the transplantation was scheduled to take place, despite having no risk factors, the patient suffered from a deep vein thrombosis (DVT) of the left lower thigh. Owing to this unusual DVT and the former flap loss mentioned above, we ran a thrombophilia diagnosis prior to the planned microvascular fibula transplantation. This diagnosis showed an APC resistance ratio of 1.43 (≥ 3.0). An APC resistance ratio of less than 3.0 suggests abnormal resistance to activated protein C, and DNA-based testing for FVL was therefore conducted, which revealed a heterozygous FVL.

### Case report 2

The second patient was a 57-year-old Caucasian male diagnosed with a pT4 pN1 L0 V0 Pn1 G2 R0 OSCC of the right mandibular angle region 45–48.

A fibula flap was used for microsurgical reconstruction. After several unsuccessful salvage attempts, the fibula transplant had to be discarded owing to recurring thrombosis during microsurgery. To ensure a sufficient defect reconstruction, a reconstruction plate covered with a pectoralis major flap was used.

After surgical treatment, the patient underwent combined radiochemotherapy.

Owing to the unusual, extensive thromboses during microsurgery, we checked the patient for possible coagulation disorders before planning a second bony reconstruction. A heterozygous FVL mutation was found, resulting in an APC resistance ratio of 1.51 (≥ 3.0).

### Case report 3

The third patient was a 56-year-old Caucasian male diagnosed with a pT4a pN0 L0 V0 Pn0 G3 R0 OSCC of the right alveolar ridge region 32–45. A fibula flap was successfully used for bony and soft tissue reconstruction. Combined radiochemotherapy was carried out.

In the following years, this patient suffered twice from locoregional recurrences. The first recurrence could be reconstructed successfully with a pectoralis major flap combined with a forearm flap owing to the large tissue defect. For the reconstruction of the second recurrence, another fibula flap from the contralateral side was successfully used. Six months later, multiple tumor recurrences led to another continuity resection of the mandible, and a two-stage reconstruction with a scapula flap was performed.

Unlike the three microvascular flaps mentioned above, this time, it was not possible to ensure constant perfusion owing to multiple, excessive, and unusual thromboses, and in the end, the flap had to be discarded. As a result, a thrombophilia diagnosis was performed, which showed a heterozygous FVL mutation with an APC resistance ratio of 1.46 (≥ 3.0).

## Modified anticoagulation protocol

### Preoperative identification of high-risk patients

After the experience with these three cases, the need for a preoperative identification of these high-risk patients arose. Ideally, high-risk patients should be detected preoperatively; however, no effective risk assessment tool is yet available. Therefore, we developed a modified questionnaire based on that of Friedman *et al*. For reasons of feasibility, we reduced the questionnaire to three questions, which we now ask in addition to inquiring about the patient’s medical history (see Table 3) [7].

**Table 3:** Modified questionnaire for thrombosis risk based on Friedman *et al*.

	Yes	No
Have you or has anyone in your family ever had a blood clot?		
Have you or has anyone in your family ever been diagnosed with a blood clotting disorder?		
For female patients: have you ever had a miscarriage?		

If the patient answers at least one question with yes, the surgeon determines the risk of thrombosis in a personal conversation with the patient on the basis of a list of risk factors [7].

If the patient is not identified as being at high risk, we proceed with our standard anticoagulation protocol for microsurgical approaches (Table 1).

If the patient is classified as being at high risk, we proceed with our modified anticoagulation protocol.

### Pannucci’s anticoagulation protocol

Pannucci *et al*.’s approach begins with an intraoperative heparin perfusion of typically 800 U/hour. Just before flap dissection, a bolus dose of 3000 U of heparin is administered. During anastomosis, a continuous heparin perfusion with a goal partial thromboplastin time (PTT) of 50–70 seconds is applied. Therapeutic heparin anticoagulation is continued during hospitalization and is transitioned to a therapeutic dose of enoxaparin (1.5 mg/kg per day or 1 mg/kg twice a day) for 4 weeks postsurgery [8].

Since this protocol was developed for plastic surgery in general, we adapted it to our special needs in head and neck cancer microsurgery based on our experience (Table 4).

**Table 4:** Our modified anticoagulation protocol for high-risk patients

Timing		Drugs	Bodyweight			Duration
			50–69 kg	70–89 kg	> 90 kg	
Preoperative	Inpatient stay (normal ward)	Antithrombin level assessment				
	Inpatient stay (normal ward)	Enoxaparin subcutaneous	30 mg/day* (prophylactic dose)	40 mg/day* (prophylactic dose)	50 mg/day* (prophylactic dose)	
Intraoperative	Beginning of surgery	Tracheotomy (when needed)				
	After tracheotomy	Heparin intravenous	600 U/hour	800 U/hour	1000 U/hour	During surgery
	Just before flap dissection	Heparin intravenous	Bolus of 2000 U	Bolus of 3000 U	Bolus of 4000 U	
	End of microsurgery	Heparin intravenous	Bolus of 1500 U	Bolus of 1500 U	Bolus of 2000 U	
Postoperative	Arrival at intensive care ward	Heparin intravenous	Continuous perfusion; goal: PTT 55–70 seconds			
		Alternative: enoxaparin subcutaneous	60 mg*	80 mg*	100 mg*	
	Inpatient stay (normal ward) / postdischarge	Heparin intravenous	Continuous perfusion; goal: PTT 55–70 seconds			Until hospital discharge
		Alternative: Enoxaparin subcutaneous	60 mg twice/day* (therapeutic dose)	80 mg twice/day* (therapeutic dose)	100 mg twice/day* (therapeutic dose)	Up to 4 weeks postsurgery

*Dose reduction for 30–50% in cases of GFR < 30 ml/minute, determination of factor Xa level

### Our modified anticoagulation protocol for high-risk patients

To ensure a safe airway management in head and neck tumor surgery, a tracheotomy is often necessary. As the presented protocol causes an increased risk of bleeding, we perform the tracheotomy first. After the tracheotomy, we begin weight-adjusted heparin perfusion that lasts the entire duration of the surgery. Just before the flap is dissected, we apply the first weight-adjusted heparin bolus. At the end of microsurgery, we apply another weight-adjusted heparin bolus.

**Fig. 1:**
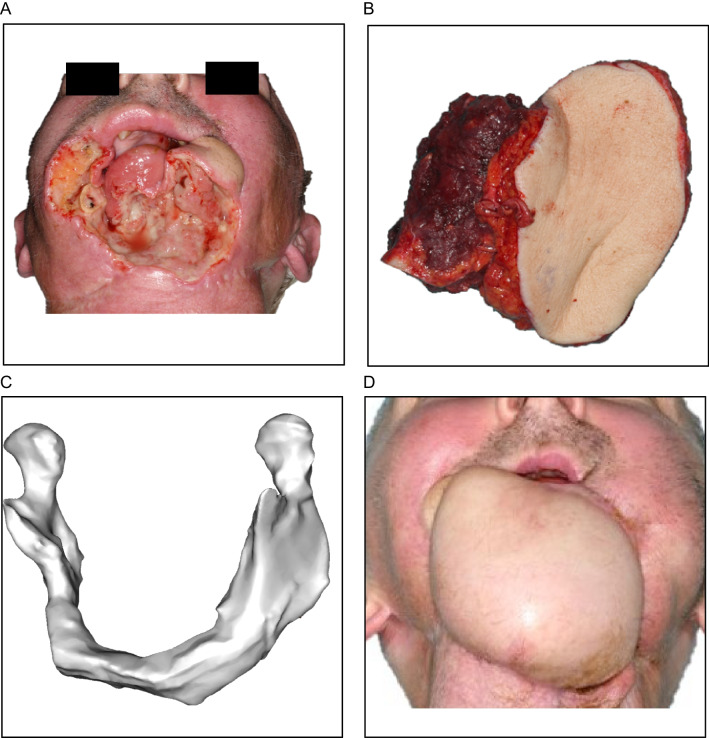
Successful microvascular transplant in Patient 3 following our anticoagulation protocol. **A** Situation after loss of the free flap and the resulting bony and soft tissue defect. **B** Combined microvascular scapula and latissimus dorsi flap. **C** Three-dimensional model of the reconstructed jaw. **D** Postoperative situation after six months

On arrival at the intensive care ward, the heparin perfusion continues and is adjusted to a goal PTT of 55–70 seconds. Alternatively, the use of a weight-adjusted, therapeutic dose of enoxaparin twice a day is possible. Therapeutic anticoagulation is continued for 4 weeks postsurgery. Heparin perfusion is switched to enoxaparin no later than at discharge. Figure 1 presents the successful microvascular transplant following the anticoagulation protocol.

## Discussion

Microvascular surgery in patients with head and neck tumors is a standard procedure with a predictable outcome. In patients with thrombophilia, however, the risk of free flap loss is significantly increased with salvage rates tending toward zero [9–11]. Thus far, no easily applicable protocol for microsurgical approaches in head and neck surgery for these high-risk patients exists.

These cases with differing clinical courses are presented to clarify the problems and pitfalls of hidden thrombophilia in daily practice. In all cases, the failure of microsurgical anastomosis was the reason for flap loss. In the presented patient collective, patient 1 presents a case with clinical thrombophilic symptoms, such as DVT, which could hint at an underlying thrombophilia. Patients 2 and 3 demonstrate the problem of a typical, unobtrusive patient, for whom flap loss owing to massive thrombosis represents the first appearance of an underlying thrombophilia. In the case of patient 3, this flap loss occurred even after multiple successful microvascular surgeries had been performed.

To ensure successful microsurgery on these high-risk patients, we developed our modified anticoagulation protocol on the basis of the approach by Pannucci *et al*. [8].

By using this modified anticoagulation protocol, we were able to successfully perform microsurgery on all three patients.

The first step of sufficient thrombosis prophylaxis begins preoperatively by assessing the patient’s individual risk of thrombosis. In contrast to Friedman *et al*., we do not run an elaborated blood test for thrombophilia before surgery as this diagnosis would take at least 2 weeks and thereby cause a delayed start of treatment [7]. However, Friedman *et al*.’s approach of assessing the patient’s risk with a simple questionnaire seems to be a valid tool for identifying these patients [7, 8]. To create an easier clinical routine, we implemented a modified questionnaire that uses only three questions. Owing to the low number of high-risk patients, a proper evaluation of this measure was not feasible.

Interestingly, an analysis of the literature yielded no generally applicable guideline for anticoagulation in microsurgery. Various heterogeneous studies recommend heparin/low-molecular-weight heparin (LMWH), aspirin, or sometimes dextran. Dextran has been noted to have a significant risk of side effects and no proven benefit in free flap survival [12]. In our protocol, we use (1) LMWH subcutaneously (sc) or (2) heparin intravenously.

In hypercoagulable patients, a systematic review by Kotamarti *et al*. found that microvascular surgery had been successful in reconstruction of the breast, lower extremities, and in only a few cases in the head and neck (86.1% success rate) [9]. The authors offer no specific anticoagulation protocol, and there are no evidence-based recommendations for drugs, their dosage, or the duration of application. However, the authors note out that beginning with intraoperative anticoagulation—which is continued for at least 5 days postoperatively using heparin with or without aspirin—seems to be successful in microvascular surgery on high-risk patients [9].

As there is no evidence in literature that aspirin improves the outcome for high-risk patients, we do not administer aspirin in microsurgery [9]. Since our success rate is similar to the reported rates in the literature, we also conclude that there is no rationale for the use of aspirin in our routine protocol.

In 2020, Nguyen *et al*. performed a retrospective study of 147 microvascular flaps in head and neck surgery in 117 patients and evaluated the success rate of microsurgery in hypercoagulable patients. In 24 patients with 28 free flaps, a hypercoagulable status was identified preoperatively. The overall success rate of flap survival was 100%, although complications occurred in 12/28 hypercoagulable patients. The authors also indicated that the anticoagulation regime varied within their study as no general protocol exists in the literature. Based on their results, Nguyen *et al*. now use 650 U/hour of intravenous heparin postoperatively in hypercoagulable patients [13].

Instead of using a steady rate of heparin perfusion, the heparin dosage in our protocol is based on bodyweight. The literature has revealed that weight-based heparin is superior to normal heparin dosages and carries the same risk of bleeding [14–17].

Postoperatively, our patients stay in the intensive care unit (ICU) before they are transferred to the normal ward the day thereafter. Herein, a continuous heparin perfusion is administered with the goal PTT of 55–70 seconds, which is continued until hospital discharge. At discharge, drug and application (heparin iv) are switched to enoxaparin sc.

Recently, enoxaparin, instead of heparin perfusion, has been administered even during hospital stay. Regarding bleeding events, enoxaparin has been shown to be equal to or even safer than heparin [18, 19]. The routine application in an ICU is intravenous heparin, which takes at least 10 hours to reach the PTT goal. This application is usually switched to an easier applicable subcutaneous anticoagulation in the general ward. This change in application might result in a gap of the anticoagulative effect. Therefore, we changed our protocol to enoxaparin directly upon admission to the ICU [17].

Another advantage of enoxaparin lies in the fact that it is easier to handle and offers patients the possibility of administering it themselves after hospital discharge. After discharging high-risk patients, we recommend enoxaparin sc for 4 weeks postsurgery. Then, a sufficient supply via peripheral revascularisation should be secured, even if primary anastomosis has failed [8].

We assess the antithrombin (AT) level and activity preoperatively, as heparin is dependent on adequate AT level and activity [20–22]. AT deficiency can be inherited or acquired, for example, owing to compromised liver synthesis [23]. Knowing the AT level and activity in advance helps to specify the perioperative dosage of heparin more precisely. An AT substitution should be considered in case of an ineffective heparin administration.

A limitation of this study is the low number of patients. Thrombophilia is often asymptomatic, and therefore, preoperative identification of high-risk patients is very difficult. Given a prevalence of around 10% for a thrombophilic disorder in the microvascular patient population, flap loss owing to thrombophilia should be observed far more often than it is reported in the literature and clinical experience. It seems that most patients with thrombophilia are treated sufficiently by using a “normal” anticoagulation protocol in microsurgery. In cases of a known and/or symptomatic thrombophilia, the modified anticoagulation protocol presented seems to be an effective tool for successful microsurgery in high-risk patients. Further studies are necessary to verify this regime.

## Conclusion

Successful head and neck microsurgery on high-risk patients suffering from thrombophilia is highly complicated. Identifying these high-risk patients preoperatively is difficult, and no perioperative anticoagulation protocol yet exists for microvascular surgery in the head and neck for these patients. We presented an easily applicable protocol for perioperative anticoagulation in this special patient population. By using this protocol, successful microvascular surgery on high-risk patients is possible.

## Data Availability

The datasets during and/or analyzed during the current study available from the corresponding author on reasonable request.
